# HDCA Supplementation During Maternal High-Fat Diet Exposure Is Associated with Offspring Gut Microbiota at Weaning and Adult Metabolic Phenotypes in a Mouse Model

**DOI:** 10.3390/metabo16080590

**Published:** 2026-08-19

**Authors:** Halizere Simayi, Yating Yang, Li Gao, Bin Yu, Yuwen Shi, Ningxin Chen, Jialing He, Meng Duan, Wei He, Shankuan Zhu, Fei Yang

**Affiliations:** 1 Department of Endocrinology of the Second Affiliated Hospital of Zhejiang University School of Medicine, Chronic Disease Research Institute, School of Public Health, School of Medicine, Zhejiang University, Hangzhou 310058, China; 3180102244@zju.edu.cn (H.S.); yangyating@zju.edu.cn (Y.Y.); 18324354104@163.com (L.G.); 3210100363@zju.edu.cn (B.Y.); 2418009@zju.edu.cn (Y.S.); 22318289@zju.edu.cn (N.C.); hejialing@zju.edu.cn (J.H.); 2 Department of Nutrition and Food Hygiene, School of Public Health, School of Medicine, Zhejiang University, Hangzhou 310058, China; zjuhewei@zju.edu.cn; 3 Binjiang Institute of Zhejiang University, Hangzhou 310058, China; 4 Children’s Hospital, Zhejiang University School of Medicine, National Clinical Research Center for Children and Adolescents’ Health and Diseases, Hangzhou 310058, China; duanmeng@zju.edu.cn; 5 Chronic Disease Research Institute, The Children’s Hospital, National Clinical Research Center for Children and Adolescents’ Health and Diseases, School of Public Health, School of Medicine, Zhejiang University, Hangzhou 310058, China

**Keywords:** maternal diet, gut microbiota, high-fat diet, offspring development, intergenerational microbial similarity

## Abstract

**Background/Objective**: Maternal diet is an important determinant of gut microbiota composition in dams and offspring. This study investigated whether HDCA supplementation during maternal high-fat diet (HFD) exposure was associated with gut microbiota composition in dams and offspring and with selected obesity-related phenotypes. **Methods**: Nineteen C57BL/6J female mice were assigned to a control diet group (CON), a high-fat diet group (HFD), and a high-fat diet supplemented with 0.5% hyodeoxycholic acid group (HFD+HDCA). Fecal samples were collected from the dams before mating, following 8 weeks of dietary intervention, and from the offspring at weaning. All samples were analyzed using 5R 16S rRNA gene sequencing. Body weight was monitored in both dams and offspring, and liver histology was assessed by hematoxylin and eosin staining. **Results**: HFD exposure was associated with obesity-related phenotypes in dams and offspring, including increased maternal body weight and greater hepatic lipid accumulation and visceral adiposity in offspring. LEfSe and ANCOM-BC2 analyses identified concordant microbial changes between dams and offspring under corresponding dietary conditions. *Lachnospiraceae_Unknown_genus3261* and *Coprococcus* increased with HFD exposure in both dams and offspring, whereas *Bifidobacterium*, *Coprococcus*, and *Allobaculum* decreased following HDCA supplementation. These findings indicate maternal–offspring concordance in microbiota responses to HFD and HDCA, without establishing direct vertical transmission. PICRUSt2 analysis suggested group association differences in predicted functional potential for pathways annotated to propionate, pyruvate, and β-alanine metabolism. **Conclusions**: HDCA supplementation during maternal HFD exposure was associated with differences in offspring gut microbiota at weaning and with attenuation of selected obesity-related phenotypes. These findings suggest a potential role of the gut microbiota–bile acid axis in mediating intergenerational dietary effects. However, the study does not establish direct microbial transmission, altered metabolic activity, or causality; these findings require confirmation in litter-aware and mechanistic studies.

## 1. Introduction

In recent decades, obesity has become increasingly prevalent among women of childbearing age, emerging as a growing global health concern [1]. Nearly half of reproductive-age women in the United States are currently classified as overweight or obese [2]. In China, the overall prevalence of overweight and obesity among childbearing-age females was 24.8% between 2010 and 2014 [3]. High intake of saturated fat is a major contributor to obesity, and animal studies have consistently shown that a high-fat diet (HFD) significantly increases body weight and white adipose tissue mass in mice [4,5].

The Developmental Origins of Health and Disease (DOHaD) framework proposes that, in addition to genetic factors, early-life environmental exposures, including maternal factors, contribute to health and disease risk in adulthood [6]. Accumulating evidence implicates maternal obesity as an important determinant of offspring health during childhood and later life. Elevated maternal weight is associated with adverse offspring outcomes, including obesity [7], cardiovascular complications [8], type 2 diabetes [9], neurodevelopmental disorders [10] and asthma [11]. Animal studies have also linked prenatal exposure to a maternal HFD with higher body weight, impaired glucose regulation, and altered lipid metabolism in offspring [1]. Nevertheless, the biological mechanisms underlying these intergenerational associations remain incompletely understood.

Diet is a major determinant of gut microbial composition and function. HFD can alter the relative abundance of taxa with diverse, context-dependent associations with host health [12]. Maternal diet may influence both the maternal gut microbiota and microbial composition in offspring. Previous studies have shown that HFD exposure before and during pregnancy can reduce the abundance of *Lactobacillus* in offspring and alter microbiota-associated inflammatory features [13]. The gut microbiota has been implicated in body weight regulation and metabolic homeostasis through effects on energy harvest, microbial metabolite production, and host signaling pathways [14]. Maternal microbial communities may contribute to offspring microbial composition through exposure during birth and lactation, maternal immune regulation [15], and microbial metabolites [16]. However, shared taxa between dams and offspring do not, by themselves, establish direct vertical transmission.

Bile acids can influence the composition and metabolic potential of the gut microbiota [17], and interactions between bile acids and intestinal microorganisms have been implicated in metabolic diseases. Hyodeoxycholic acid (HDCA) is a natural secondary hydrophilic bile acid abundant in pig and rat bile [16]. Oral HDCA administration has been reported to ameliorate non-alcoholic fatty liver disease (NAFLD) phenotypes in HFD-fed mice, accompanied by enhanced lipid catabolism [18]. However, whether HDCA supplementation during maternal HFD exposure is associated with microbial alterations in both dams and offspring, and whether these alterations accompany changes in offspring obesity-related phenotypes, remains unclear.

Therefore, this study aimed to determine whether HDCA supplementation during maternal HFD exposure was associated with (i) differences in gut microbiota composition in dams, (ii) concordant taxonomic patterns in offspring at weaning, and (iii) changes in offspring obesity-related phenotypes later in life. To address these objectives, we combined body- and liver-weight measurements and descriptive liver histology with 5R 16S rRNA gene sequencing and PICRUSt2-based functional prediction.

## 2. Materials and Methods

All experimental procedures were approved by the Animal Care and Use Committee of Zhejiang University (approval number: ZJU 20240781).

### 2.1. Animals and Housing

Four-week-old male and female C57BL/6J mice (body weight 12–18 g) were purchased from Shanghai Slack Laboratory Animal Co., Ltd. (Shanghai, China). Mice were housed in a specific pathogen-free (SPF) environment (22.5 ± 2 °C, 12 h light/dark cycle, 55 ± 15% humidity) with ad libitum access to food and water.

### 2.2. Experimental Design

After one week of acclimation, 5-week-old female mice were randomly assigned to three groups based on body weight to minimize baseline differences among groups: the control (CON, *n* = 6) group fed a standard diet (catalog no. LAD3001G), the high-fat diet (HFD, *n* = 6) group fed an HFD (catalog no. TP23520), and the high-fat diet supplemented with 0.5% hyodeoxycholic acid (HFD+HDCA, *n* = 7). The HFD+HDCA diet was custom-prepared by the same manufacturer by incorporating 0.5% HDCA into the HFD formulation. This concentration was selected based on our previous study demonstrating that dietary HDCA supplementation at a comparable dose range exerted beneficial metabolic effects in mice [19,20]. Therefore, 0.5% HDCA was selected as a biologically relevant dose for evaluating its potential protective effects under maternal HFD exposure. All diets were purchased from Nantong Trophic Animal Feed High-tech Co., Ltd. (Nantong, Jiangsu, China). and gamma-irradiated. Detailed nutritional compositions are provided in Supplementary Table S1. Food intake was measured weekly at the cage level. The amount of consumed diet per cage was calculated based on the difference between provided and remaining food and was normalized to the number of mice housed in each cage. Following 8 weeks of dietary intervention, when diet-induced differences in body weight had emerged and stabilized, fecal samples were collected from each female mouse one day before mating for gut microbiota analysis. Female mice were maintained on their assigned CON, HFD, or HFD+HDCA diets throughout mating, pregnancy, and lactation.

Female mice were then mated with age-matched 13-week-old male mice at a ratio of two females to one male. Male mice were maintained on a standard diet before mating. During the mating period, male mice were housed in the same cages as the female mice and were provided with the same diet as the paired females (CON, HFD, or HFD+HDCA diet). The male mice used for mating were of similar age and body weight across groups and were randomly assigned to each dietary group. Successful mating was confirmed by the presence of a vaginal plug, after which male mice were removed from the cages, and pregnant females were maintained on their assigned diets until delivery. All dams were allowed to deliver naturally. Offspring were weaned at postnatal day 21 (PND 21), separated by sex, and fed a standard diet until 20 weeks of age. Fecal samples were collected from offspring at PND 21, immediately after weaning, for gut microbiota analysis. The offspring groups were designated as GCON, GHFD, and GHFD+HDCA, corresponding to offspring born to dams assigned to the CON, HFD, and HFD+HDCA groups, respectively. Thus, CON/HFD/HFD+HDCA represented the maternal groups, whereas GCON/GHFD/GHFD+HDCA represented the corresponding offspring groups. The numbers of offspring analyzed were 17 (9 males and 8 females) in the GCON group, 11 (6 males and 5 females) in the GHFD group, and 10 (5 males and 5 females) in the GHFD+HDCA group. The sex distribution was approximately balanced across dietary groups. The differences in offspring sample sizes were due to natural variations in litter size and occasional pup mortality during lactation. Maternal mice were euthanized after weaning. Offspring body weight was recorded weekly from PND 21 to 20 weeks of age. At the experimental endpoint, the offspring were euthanized at 20 weeks of age, and liver samples were collected for subsequent analyses. All animal procedures were conducted in accordance with the ARRIVE guidelines.

### 2.3. DNA Extraction and 5R 16S rRNA Gene Sequence Analysis

Fecal samples (40–70 mg) were subjected to total genomic DNA extraction using the CTAB method with a bacterial DNA extraction kit (DP302-02, Tiangen, Beijing, China). The 5R 16S rRNA gene sequencing strategy was employed, which simultaneously amplifies five hypervariable regions (V2, V3, V5, V6, and V8) of the 16S rRNA gene via multiplex PCR. This approach covers approximately 68% of the full-length 16S rRNA gene, improving taxonomic resolution, especially for low-biomass samples. PCR products were purified and quantified. Libraries were prepared and sequenced on an Illumina NovaSeq 6000 platform (PE150) (Illumina, Inc., San Diego, CA, USA). Raw sequencing data were processed using the SMURF (Short MUltiple Regions Framework) pipeline for taxonomic classification. Potential contaminant sequences introduced during sampling, DNA extraction, or PCR amplification were removed based on negative controls (air blanks, extraction blanks, and no-template controls).

The following parameters were applied for sequencing data quality control and processing: raw reads were filtered using fqtrim software (version 0.94) to remove adapter sequences and reads with an average quality score < Q20, and low-quality bases at the 3′ ends were trimmed. The effective read count per sample ranged from 130,000 to 210,000, with mean Q20 and Q30 values of 98.7% and 95.8%, respectively. Taxonomic assignment was performed using the optimized Greengenes database (May 2013 release) through the SMURF (Short MUltiple Regions Framework) pipeline, which integrates sequence information from five amplified regions for OTU generation and taxonomic identification. To account for differences in sequencing depth across samples, rarefaction was applied, with all samples rarefied to 65,219 reads prior to diversity analyses. Rarefaction was applied only for diversity analyses, whereas relative abundance data were used for taxonomic composition and differential abundance analyses. OTUs with a total relative abundance <0.005% across all samples were filtered out. Negative controls, including sampling blanks, DNA extraction blanks, and no-template PCR controls, yielded very few sequences, mainly affiliated with environmental genera such as *Sphingomonas* and *Acinetobacter*, accounting for <0.1% of total effective reads in each control. These potential contaminant sequences were removed before downstream analyses.

### 2.4. Histological Staining

Liver tissues were collected, fixed in 4% paraformaldehyde, embedded in paraffin, sectioned at a thickness of 4 µm, and stained with hematoxylin and eosin (H&E). Images were captured using an Olympus VS200 digital slide scanner (Olympus Corporation, Tokyo, Japan) at 200× magnification (scale bar, 20 µm).

### 2.5. Statistical Analysis

Alpha diversity (Shannon and Simpson indices) was calculated to assess microbial diversity and community evenness. Differences among groups were assessed by the Kruskal–Wallis test, followed by the Wilcoxon rank-sum test for pairwise comparisons with Bonferroni correction.

Beta diversity was evaluated using principal coordinate analysis (PCoA) based on Bray–Curtis distance. PERMANOVA (adonis) with 999 permutations was used to test significant differences between groups, and pairwise comparisons were adjusted by Bonferroni correction.

Taxonomic composition was analyzed at the phylum, family, and genus levels, and relative abundances were visualized as stacked bar plots.

Linear discriminant analysis Effect size (LEfSe) was applied to identify differentially abundant genera, with thresholds set at LDA score > 2.5 and *p* < 0.05. ANCOM-BC2 was subsequently performed as an independent differential abundance analysis to validate the taxa identified by LEfSe. Differential abundance analysis was performed separately for maternal and offspring samples. Maternal groups were compared as CON vs. HFD and HFD vs. HFD+HDCA, whereas offspring groups were compared as GCON vs. GHFD and GHFD vs. GHFD+HDCA. To evaluate intergenerational concordance, microbial taxa identified in corresponding maternal and offspring dietary groups were subsequently compared descriptively, including CON–GCON, HFD–GHFD, and HFD+HDCA–GHFD+HDCA.

Functional potential of the gut microbiota was predicted using PICRUSt2 with default parameters based on 16S rRNA gene sequencing data. KEGG Ortholog (KO) abundances were inferred, and pathway-level comparisons between groups were performed using STAMP software (version 9.0, two-sided *t*-test, *p* < 0.05).

Longitudinal body-weight data were analyzed using two-way repeated-measures ANOVA for maternal mice and linear mixed-effects models for offspring, with group, time, sex, and their interactions included as fixed effects and individual mouse identity as a random effect. For other phenotypic data, including liver weight and relative liver weight, one-way ANOVA followed by Tukey’s post hoc test was used for multiple comparisons. Student’s *t*-test was applied for two-group comparisons. Non-normally distributed microbiota data were analyzed using Kruskal–Wallis and Wilcoxon tests with Bonferroni correction. Data are presented as mean ± SEM. Statistical analyses and graphical visualization were performed using GraphPad Prism (version 9.5.0) and R Studio (version 4.4.2). Statistical significance was set at *p* < 0.05.

## 3. Results

### 3.1. HDCA Supplementation Alleviates HFD-Induced Obesity-Related Phenotypes

To determine whether HFD induced adverse effects and whether HDCA alleviated these effects, body weight and liver weight were assessed in dams and offspring across the three dietary conditions.

**Figure 1:**
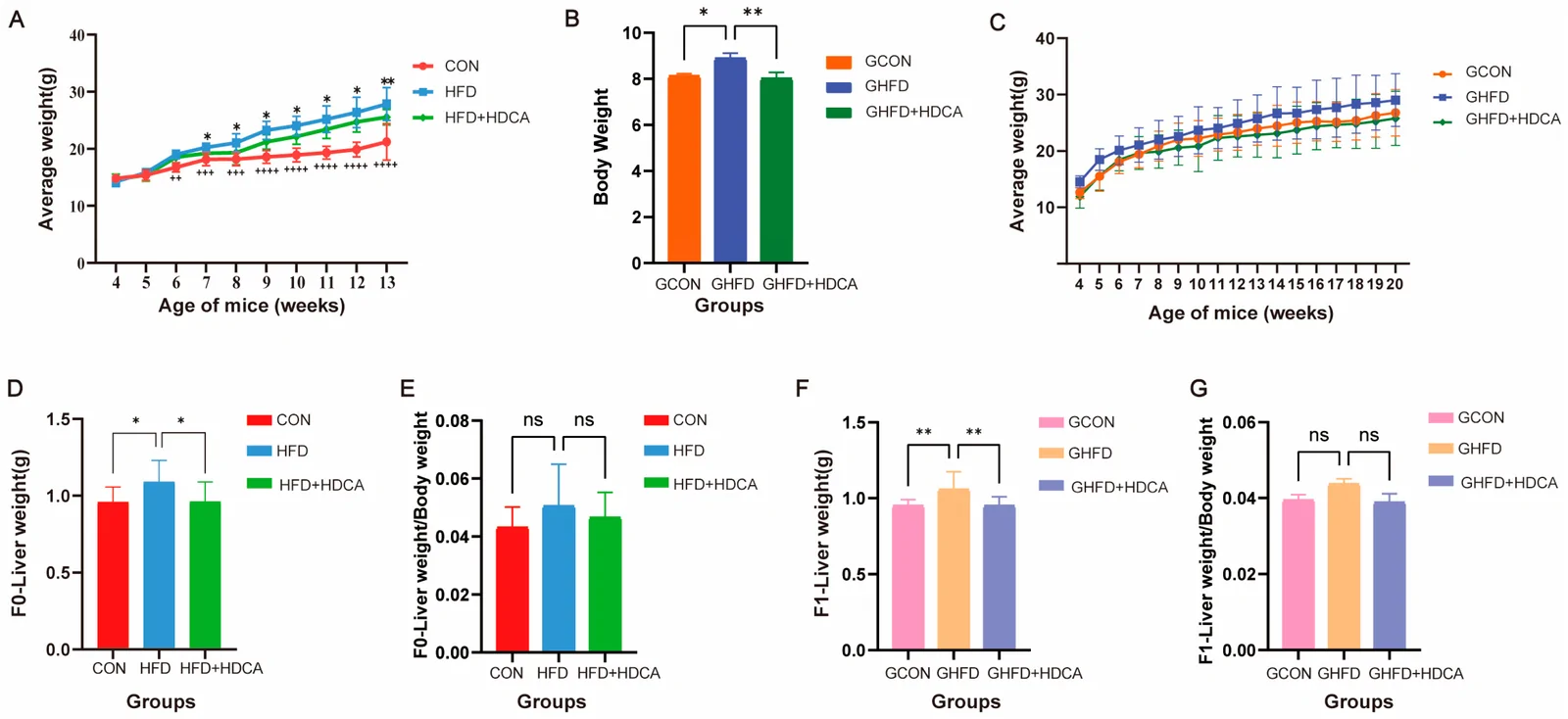
Effects of maternal dietary interventions on body weight and liver weight in dams and offspring. (**A**) Maternal body weight during 8-week intervention. (**B**) Offspring body weight at weaning (3 weeks). (**C**) Offspring body weight from weaning to 20 weeks. (**D**) Absolute liver weight of dams. (**E**) Relative liver weight of dams normalized to body weight. (**F**) Absolute liver weight of offspring. (**G**) Relative liver weight of offspring normalized to body weight. In panel A, * indicates comparison between the HFD and HFD+HDCA groups, and + indicates comparison between the HFD and CON groups. ns: no significance, * *p* < 0.05, ** *p* < 0.01, ++ *p* < 0.01, +++ *p* < 0.001, ++++ *p* < 0.0001. CON, control diet; HFD, high-fat diet; HFD+HDCA, high-fat diet with HDCA; GCON, GHFD, GHFD+HDCA, corresponding offspring groups.

Maternal body weight was monitored weekly for 8 weeks (from 5 to 13 weeks of age) (Figure 1A). Two-way repeated-measures ANOVA revealed significant effects of dietary group, time, and the group-by-time interaction on maternal body weight (all *p* < 0.0001; Supplementary Table S2). The HFD group exhibited progressively increased body weight compared with the CON group and the HFD+HDCA group throughout the intervention period. At the end of the intervention (13 weeks of age, before mating), the mean body weight was 21.22 ± 3.19 g in the CON group, 27.86 ± 2.86 g in the HFD group, and 25.54 ± 1.34 g in the HFD+HDCA group. The HFD group showed significantly higher body weight than both the CON group (*p* < 0.0001) and the HFD+HDCA group (*p* < 0.01). To determine whether the metabolic effects of HDCA supplementation were influenced by differences in energy intake, cage-based food consumption was monitored weekly and converted to energy intake based on the caloric density of each diet. The HFD+HDCA group did not show reduced energy intake compared with the HFD group; rather, it exhibited higher energy intake during the intervention period (Supplementary Figure S1). These findings suggest that the beneficial effects of HDCA supplementation were unlikely to be solely explained by reduced food intake.

Offspring body weight was monitored from weaning (3 weeks of age) to 20 weeks of age (Figure 1B,C). The GHFD group had significantly higher body weight than both the GCON and GHFD+HDCA groups (*p* < 0.05). Longitudinal body weight data were analyzed using a linear mixed-effects model with diet group, sex, and time as fixed effects and individual mouse identity as a random effect. Significant effects of group, sex, and age were observed (*p* = 0.0062, *p* < 0.001, and *p* < 0.0001, respectively), and a significant group-by-time interaction was detected (*p* = 0.0058), indicating different body-weight trajectories among offspring groups (Supplementary Table S3). No significant group-by-sex interaction was observed (*p* = 0.260), suggesting that the effects of maternal dietary intervention on offspring body weight were not sex-dependent. Although the GHFD group showed a tendency toward higher body weight after weaning, no significant differences among the three offspring groups were detected during adulthood.

Regarding liver weight (Figure 1D–G), the absolute liver weights of HFD dams and GHFD offspring were significantly higher than those of their respective control groups (CON and GCON, *p* < 0.05 and *p* < 0.01, respectively). Similarly, HFD+HDCA and GHFD+HDCA groups showed reduced absolute liver weights compared with their corresponding HFD groups. However, after normalization to body weight, no significant differences in relative liver weight were observed among the groups. These results indicate that the differences in absolute liver weight may be partially associated with overall body size differences.

**Figure 2:**
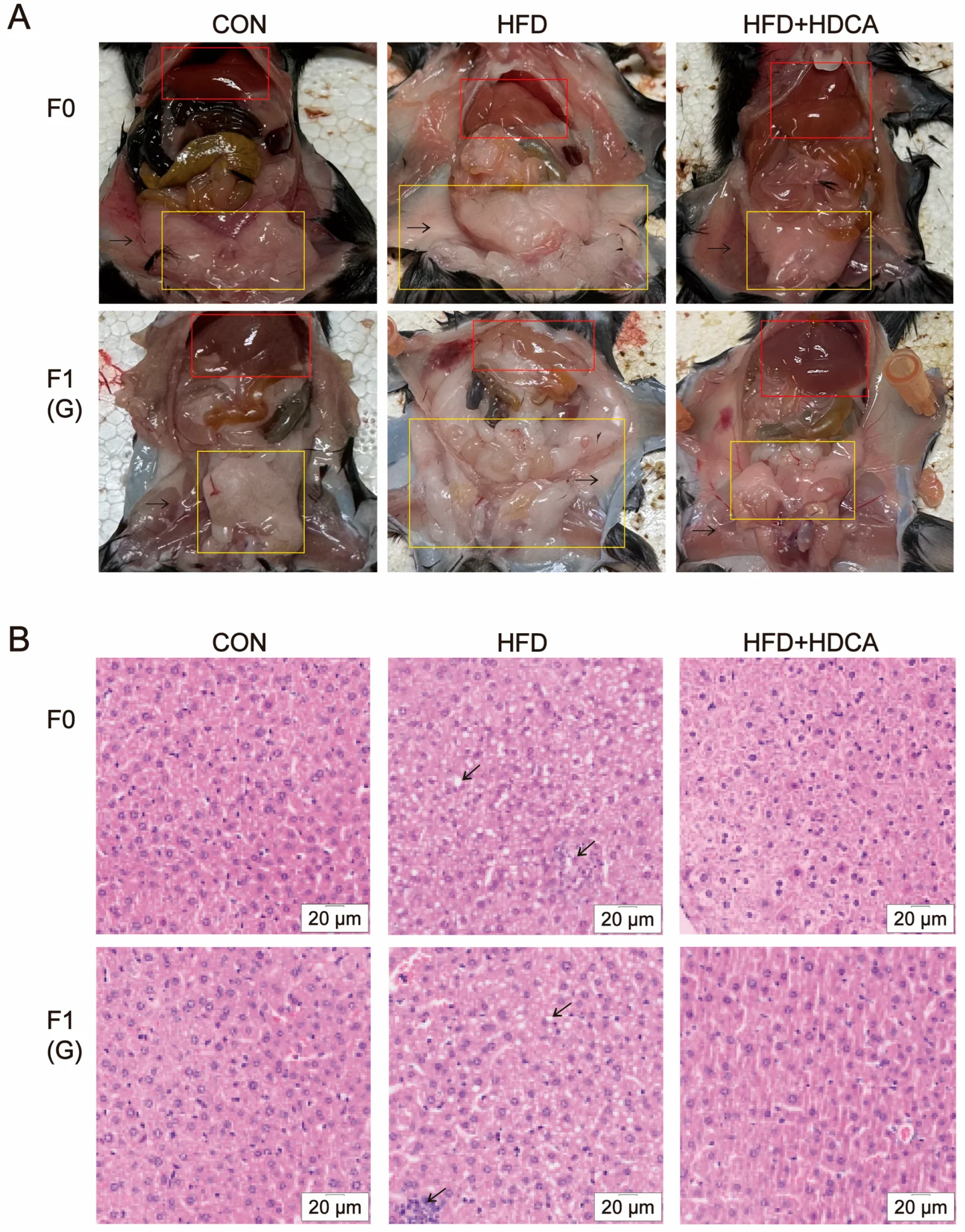
Effects of maternal dietary interventions on visceral fat distribution and liver histology. (**A**) Gross anatomy of visceral fat. Red brackets denote mouse livers, yellow brackets denote retroperitoneal fat pads, and black arrows indicate inguinal fat pads. (**B**) H&E staining of liver sections (scale bar = 20 µm). Representative images from each group are shown. CON, control diet; HFD, high-fat diet; HFD+HDCA, high-fat diet supplemented with 0.5% hyodeoxycholic acid; GCON, GHFD, GHFD+HDCA, corresponding offspring groups.

To further evaluate whether maternal dietary interventions influenced adipose distribution and hepatic morphology in dams and offspring, gross anatomical examination and histological analysis were performed. Gross anatomical observation of visceral fat distribution revealed that the CON and GCON groups had extremely thin retroperitoneal and inguinal fat pads, no obvious lipid accumulation, and normal red-brown livers (Figure 2A). In contrast, the HFD and GHFD groups exhibited abundant soft, milky-white, and expandable adipose tissue in subcutaneous and inguinal regions, with mesenteric and visceral fat significantly thickened and wrapping around the organs, accompanied by enlarged livers with blunt edges and pale-yellow discoloration. On gross examination, the HFD+HDCA and GHFD+HDCA groups appeared to have reduced fat deposition, and their liver morphology more closely resembled that of the control diet groups, as judged from the representative gross images.

Histological examination of liver sections by H&E staining showed that the CON and GCON groups had normal hepatocyte morphology with no obvious lipid droplets or inflammatory cell infiltration (Figure 2B). In contrast, the HFD and GHFD groups displayed numerous large lipid droplets within hepatocytes, accompanied by inflammatory cell infiltration. The HFD+HDCA and GHFD+HDCA groups exhibited fewer lipid droplets and reduced inflammatory cell infiltration compared with the corresponding HFD groups, with hepatic morphology showing a tendency toward improvement.

These findings suggest that maternal HDCA supplementation effectively mitigates high-fat diet-induced intra-abdominal and visceral fat accumulation, reduces hepatic steatosis, and helps partially restore liver morphology toward a normal state in both dams and offspring.

### 3.2. Gut Microbial Diversity and Community Structure

**Figure 3:**
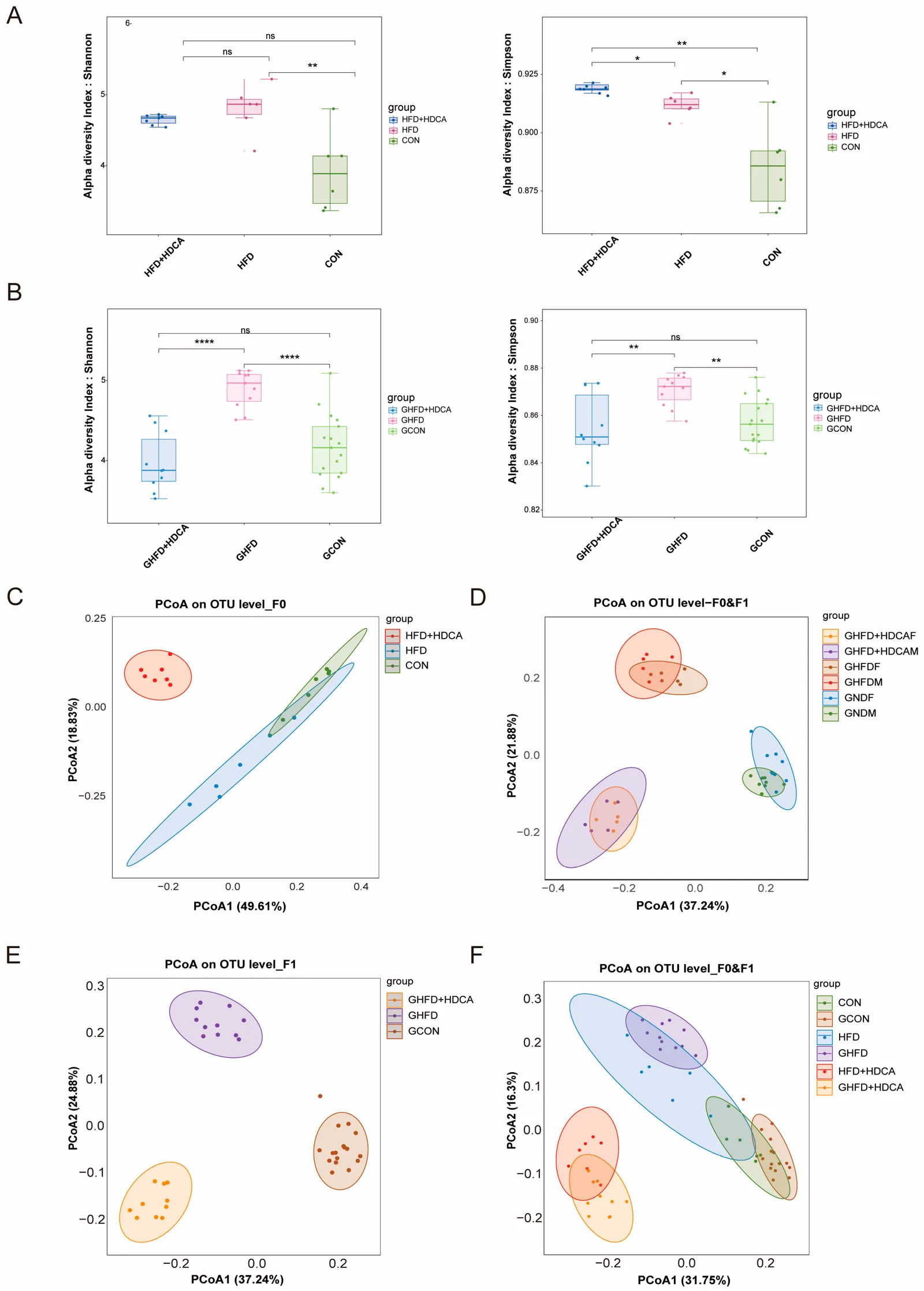
Gut microbial diversity and community structure. (**A**) Alpha diversity indices for the maternal generation. (**B**) Alpha diversity indices for the offspring generation. * *p* < 0.05, ** *p* < 0.01, **** *p* < 0.0001; ns, not significant. (**C**) Principal coordinate analysis (PCoA) plot based on Bray–Curtis distances showing gut microbial community structure in maternal mice (CON, HFD, HFD+HDCA). (**D**) Sex-stratified PCoA analysis within each offspring dietary group (GCON, GHFD, GHFD+HDCA) comparing male and female offspring. (**E**) PCoA plot for offspring mice at weaning (GCON, GHFD, GHFD+HDCA). (**F**) Combined PCoA analysis of dams and offspring (CON, HFD, HFD+HDCA, GCON, GHFD, GHFD+HDCA). CON, control diet; HFD, high-fat diet; HFD+HDCA, high-fat diet supplemented with 0.5% hyodeoxycholic acid; GCON, GHFD, and GHFD+HDCA denote the corresponding offspring groups; M, male; F, female. PERMANOVA was performed using Bray–Curtis distances with 999 permutations; *p* values were adjusted using the Bonferroni correction.

Alpha diversity analysis revealed associations between maternal dietary interventions and changes in microbial richness and evenness (Figure 3A). In maternal mice, the HFD group showed a higher Shannon index than the CON group (*p* < 0.01), while no significant differences were observed between the HFD and HFD+HDCA groups, nor between the CON and HFD+HDCA groups. Regarding the Simpson index, the HFD group was higher than both the CON group (*p* < 0.05) and the HFD+HDCA group (*p* < 0.05); additionally, the CON group was higher than the HFD+HDCA group (*p* < 0.01). These results suggest that HFD is associated with increased gut microbial diversity in dams, whereas HDCA intervention was associated with diversity values lower than those of the CON group, indicating a potential shift in microbial community structure rather than a simple return to the CON state.

In offspring, the GHFD group showed higher Shannon and Simpson indices than both the GCON and GHFD+HDCA groups (*p* < 0.0001) (Figure 3B). In addition, significant differences were also observed between the GCON and GHFD+HDCA groups, indicating that maternal HDCA supplementation did not fully restore microbial diversity to the control diet level.

Beta diversity analysis using principal coordinate analysis (PCoA) based on Bray–Curtis distances further illustrated differences in microbial community structure (Supplementary Table S4). Clustering patterns revealed distinct microbial community structures between dams and offspring under different dietary conditions. The CON group exhibited relatively concentrated sample distribution, whereas the HFD group showed more dispersed sample distribution. Following HDCA intervention, the distribution of the HFD+HDCA group differed from that of the HFD group and partially overlapped with the CON group in the spatial ordination (Figure 3C). Given that sex is a well-recognised factor influencing gut microbiota composition, we further performed sex-stratified analyses within each offspring dietary group to assess whether the observed dietary effects were consistent between males and females. Beta diversity analysis (PERMANOVA, based on Bray–Curtis distances) revealed a significant difference in microbial community structure between male and female mice in the normal control group (R^2^ = 0.342, adjusted *p* = 0.0025), with sex explaining 33.4% of the total variation (Figure 3D). This indicates a marked sexual dimorphism in the gut microbiota under normal physiological conditions, consistent with previous reports [21,22,23]. In contrast, no significant sex-based differences were observed in either the high-fat diet group or the HDCA-treated group (adjusted *p* > 0.05), suggesting that sex-associated variation was less evident under maternal dietary intervention in this cohort. Similarly, Lefebvre et al. [24] demonstrated that a high-fat diet can alter sex-dependent differences in gut microbiota, and Loeven et al. [25] further confirmed that dietary interventions affect microbiota composition in a sex-dependent manner, yet such sex effects may be diminished or masked under strong external perturbations.

Offspring groups (GCON, GHFD, GHFD+HDCA) showed a trend of spatial separation in the PCoA plot (Figure 3E). Control diet groups (CON and GCON) clustered relatively closely. High-fat diet groups (HFD and GHFD) displayed more scattered distribution with limited overlap between them. The HFD+HDCA and GHFD+HDCA groups exhibited some overlap in spatial distribution and were clearly separated from the HFD groups (Figure 3F).

Taken together, maternal HFD was associated with increased α-diversity in both generations, while HDCA intervention was associated with diversity values closer to those of the control diet group. β-diversity analysis indicated that HFD was associated with greater heterogeneity in microbial community structure, whereas HDCA intervention was associated with a distinct community composition that partially overlapped with the control diet group in spatial distribution. It should be noted that increased α-diversity under HFD does not necessarily indicate a healthier microbiota but may reflect expansion of opportunistic taxa or community instability.

### 3.3. Venn Diagram Analysis of Microbial Communities

**Figure 4:**
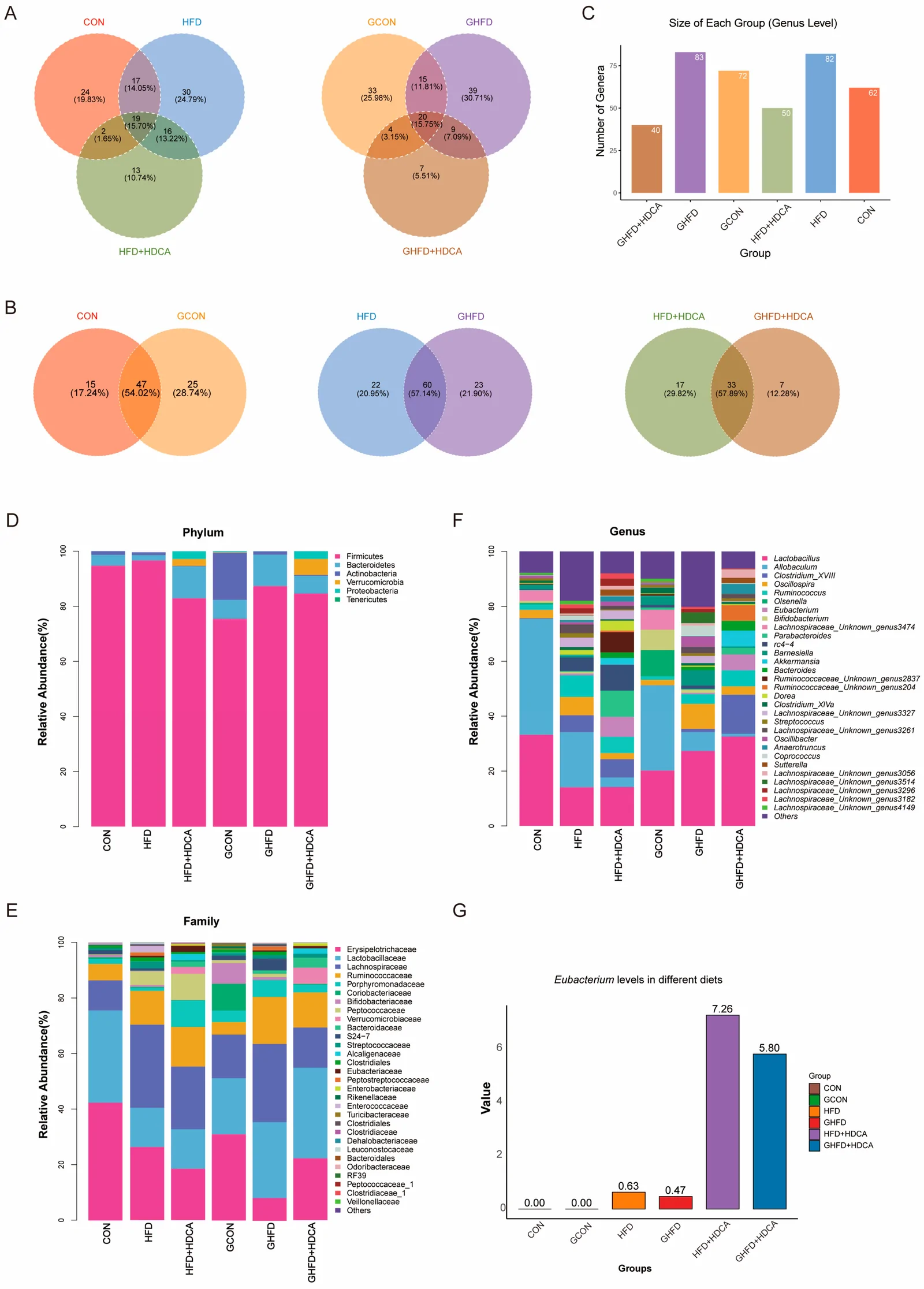
Gut microbial community composition and shared OTUs. (**A**) Venn diagrams showing shared OTUs among maternal and offspring groups. (**B**) Pairwise shared OTUs between CON–GCON, HFD–GHFD, and HFD+HDCA–GHFD+HDCA. (**C**) Characteristic OTU numbers per group. Numbers in (**A**–**C**) indicate overlapping or unique OTUs. (**D**) Relative abundance at the phylum level. (**E**) Relative abundance at the family level. (**F**) Relative abundance at the genus level. Genera with <1% abundance are grouped as “Others”. (**G**) A separate bar plot for *Eubacterium* abundance (values: HFD+HDCA = 7.26, GHFD+HDCA = 5.80; HFD = 0.63, GHFD = 0.47; CON and GCON = 0). CON, control diet; HFD, high-fat diet; HFD+HDCA, high-fat diet supplemented with 0.5% hyodeoxycholic acid; GCON, GHFD, GHFD+HDCA, corresponding offspring groups.

Venn diagram analysis revealed that 19 OTUs were shared among the three maternal groups (CON, HFD, HFD+HDCA), and 20 OTUs were shared among the three offspring groups (GCON, GHFD, GHFD+HDCA) (Figure 4A). Pairwise comparisons between dams and offspring showed that 47 OTUs were shared between dams and offspring within the same dietary group, 60 between HFD and GHFD, and 33 between HFD+HDCA and GHFD+HDCA (Figure 4B). Genus-level microbial richness of each group is further summarized in Figure 4C. Specifically, the maternal HFD group and the offspring GHFD group contained 82 and 83 genera, respectively, whereas HDCA supplementation decreased the genus counts to 50 in dams and 40 in offspring. Several bacterial genera were shared between dams and offspring. However, because maternal fecal samples were collected before mating and offspring samples were collected at weaning, these shared taxa cannot be interpreted as direct evidence of vertical microbial transmission and may also reflect shared environmental exposures or diet-associated microbial patterns.

### 3.4. Relative Abundance Analysis at Phylum, Family, and Genus Levels

At the phylum level, Firmicutes, Bacteroidetes, and Actinobacteria were the predominant bacterial phyla across all experimental groups (Figure 4D). At the family level, Erysipelotrichaceae and Lachnospiraceae were identified as the dominant families (Figure 4E). At the genus level, *Lactobacillus* and *Allobaculum* were the most abundant genera (Figure 4F). Notably, the high-fat diet with HDCA groups (HFD+HDCA and GHFD+HDCA) exhibited a marked increase in the abundance of *Eubacterium* (7.26 and 5.80, respectively), whereas this genus was not detected in the control diet groups (CON and GCON, both 0) and was present at very low levels in the high-fat diet groups (HFD: 0.63; GHFD: 0.47) (Figure 4G). *Eubacterium* is a key microbial taxon involved in secondary bile acid transformation and short-chain fatty acid (SCFA) production, and its enrichment may be associated with improved host metabolic health.

### 3.5. Differential Abundance and Maternal–Offspring Concordance of Gut Microbiota

**Figure 5:**
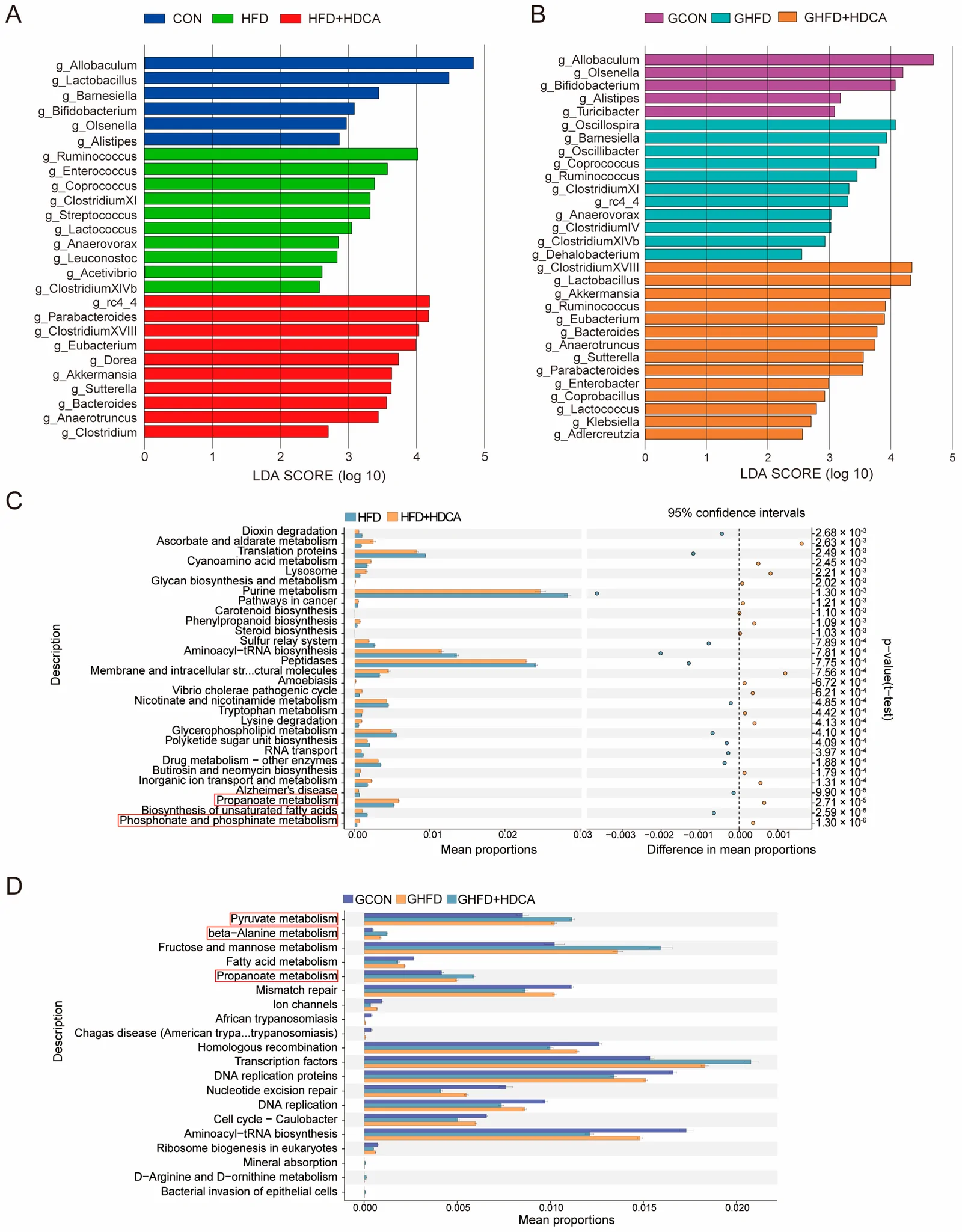
LEfSe analysis and PICRUSt2-predicted functional pathways of gut microbiota. (**A**) Linear discriminant analysis Effect Size (LEfSe) analysis showing differentially abundant bacterial genera among the three maternal groups (CON, HFD, HFD+HDCA). Only genera with LDA score >2.5 and *p* < 0.05 are shown. (**B**) LEfSe analysis showing differentially abundant bacterial genera among the three offspring groups (GCON, GHFD, GHFD+HDCA). (**C**) PICRUSt2 predicted functional pathways based on KEGG, showing differentially abundant predicted pathways in maternal mice (HFD vs. HFD+HDCA). (**D**) PICRUSt2-predicted KEGG pathways, showing differentially abundant predicted pathways in offspring mice (GHFD vs. GHFD+HDCA). Red brackets denote differentially abundant predicted pathways in maternal and offspring mice.CON, control diet; HFD, high-fat diet; HFD+HDCA, high-fat diet supplemented with 0.5% hyodeoxycholic acid; GCON, GHFD, GHFD+HDCA, corresponding offspring groups.

Linear discriminant analysis Effect size (LEfSe) was first performed to characterize differentially abundant bacterial genera among the three maternal dietary groups (CON, HFD, and HFD+HDCA) (Figure 5A). In dams, the CON group was characterized by genera including *Allobaculum*, *Bifidobacterium*, and *Lactobacillus*, whereas the HFD group was characterized by *Ruminococcus*, *Enterococcus*, *Coprococcus*, and *Clostridium* XI, among others. The HFD+HDCA group was characterized by several distinct genera, including *Parabacteroides*, *Akkermansia*, *Bacteroides*, *Anaerotruncus*, and *Clostridium* XVIII.

A similar LEfSe analysis was conducted in offspring (Figure 5B). The GCON group was characterized by *Allobaculum*, *Olsenella*, *Bifidobacterium*, and *Alistipes*. In contrast, *Coprococcus*, *Clostridium* XI, *Ruminococcus*, and *Anaerovorax* were among the genera characterizing the GHFD group, whereas *Anaerotruncus*, *Parabacteroides*, *Akkermansia*, *Bacteroides*, and *Clostridium* XVIII were enriched in the GHFD+HDCA group. Previously reported biological associations of selected genera identified in the maternal and offspring microbiota are summarized in Table 1. These associations were interpreted cautiously because genus-level biological functions may vary according to species, strain, host condition, and experimental context.

To further assess the LEfSe-derived patterns using a compositional differential-abundance approach, ANCOM-BC2 was subsequently applied to pairwise comparisons within dams and offspring. We then compared the directions of HFD-associated changes between dams (HFD vs. CON) and offspring (GHFD vs. GCON). *Lachnospiraceae_Unknown_genus3261* and *Coprococcus* showed concordant increases in both comparisons, indicating a shared HFD-associated directional pattern between the maternal and offspring microbiota. The concordant maternal–offspring changes identified by ANCOM-BC2, together with the corresponding effect estimates and adjusted *p* values, are summarized in Supplementary Table S5.

We further examined taxa showing concordant responses to HDCA supplementation by comparing HFD+HDCA versus HFD in dams with GHFD+HDCA versus GHFD in offspring. *Bifidobacterium*, *Coprococcus*, and *Allobaculum* showed decreases in both dams and offspring following HDCA supplementation. Notably, *Coprococcus* exhibited an HFD-associated increase in both dams and offspring and an HDCA-associated decrease in both comparisons, representing the clearest concordant directional response across the two datasets.

Taken together, integration of the LEfSe and ANCOM-BC2 analyses revealed several genera with concordant directional changes between dams and offspring under corresponding dietary conditions. These findings indicate similarity in microbiota responses associated with maternal HFD exposure and HDCA supplementation. However, because maternal fecal samples were collected before mating whereas offspring samples were collected at weaning, and strain-resolved source-tracking analyses were not performed, these concordant patterns should not be interpreted as evidence of persistent microbial transmission or direct vertical transmission from dams to offspring.

### 3.6. Functional Profiling of Maternal and Offspring Gut Microbiota via PICRUSt2

PICRUSt2 was used to predict the functional potential of the gut microbiota based on 16S rRNA sequencing data. Importantly, PICRUSt2 provides an inference of microbial functional potential based on phylogenetic information and does not directly measure microbial metabolic activity or metabolite concentrations. In the maternal generation, the differences in predicted metabolic pathways between the CON and HFD groups were relatively dispersed, and several pathways showed low abundance, making effective comparison difficult (Figure 5C). Therefore, subsequent pathway-level comparisons focused on the HFD and HFD+HDCA groups. In the maternal generation, compared to the HFD group, the HFD+HDCA group exhibited enrichment in two metabolic pathways related to microbial metabolism, including propionate metabolism and phosphonate metabolism.

In the offspring generation, the GHFD+HDCA group, relative to the GHFD group, showed enrichment in additional predicted pathways associated with microbial metabolism and energy-related functions, including pyruvate metabolism, propionate metabolism, and β-alanine metabolism (Figure 5D).

These findings suggest that HDCA intervention was associated with alterations in predicted microbial functional potential, including pathways related to propionate and pyruvate metabolism, in both maternal and offspring microbiota. Together with the LEfSe results showing enrichment of Eubacterium, these findings provide preliminary evidence suggesting that HDCA may influence gut microbial functional characteristics. However, because microbial metabolites, including SCFAs and bile acids, were not directly quantified in the present study, the potential involvement of microbiota-derived metabolites remains hypothetical and requires further validation using targeted metabolomic approaches.

## 4. Discussion

Previous studies have primarily described associations between HFD exposure and gut microbiota, whereas fewer studies have examined HDCA supplementation during HFD exposure in dams and offspring. The principal findings were that: (1) maternal HFD and HDCA supplementation were associated with distinct gut microbial profiles in dams; (2) corresponding offspring groups (GCON, GHFD, and GHFD+HDCA) also showed group-associated differences in gut microbial composition at weaning; and (3) several taxa showed similar or shared patterns between dams and offspring within corresponding dietary groups, particularly in the HFD and HFD+HDCA conditions. These findings are associative and should not be interpreted as evidence of persistent colonization, direct vertical transmission, or microbial inheritance.

High-fat diet exposure has been associated with alterations in the maternal and offspring gut microbiome [43,44]. In the present study, several genera showed concordant directional changes between dams and offspring under corresponding dietary conditions. ANCOM-BC2 analysis showed that *Lachnospiraceae_Unknown_genus3261* and *Coprococcus* increased with HFD exposure in both dams and offspring, whereas *Bifidobacterium*, *Coprococcus*, and *Allobaculum* decreased following HDCA supplementation in both comparisons. These findings suggest similarities in microbiota responses to HFD exposure and HDCA supplementation between dams and offspring. However, because maternal samples were collected before mating and offspring samples were collected at weaning, and strain-resolved source tracking was not performed, these concordant taxonomic patterns cannot establish direct dam-to-offspring microbial transmission. Shared environmental, dietary, litter, and cage-related exposures may also have contributed to the observed similarities. *Coprococcus* showed a particularly consistent pattern, increasing with HFD exposure and decreasing following HDCA supplementation in both dams and offspring. This concordant response suggests that *Coprococcus* may represent a microbiota feature sensitive to HFD-associated perturbation and its modification by HDCA. However, because the metabolic implications of *Coprococcus* can vary across species, strains, and host contexts, its functional significance should be interpreted cautiously.

Interaction between gut microbiota and bile acids may contribute to the observed associations. HDCA, a bile acid modified by gut microbiota, possesses multiple biological functions including promoting fat digestion/absorption, dissolving gallstones, regulating blood lipids, and improving liver function. It shows diagnostic-therapeutic potential for NAFLD treatment, glucose homeostasis modulation, and T2DM risk prediction [39]. Previous research reveals that bile acid signaling can influence host metabolism and microbial ecology [45]. The co-occurrence of *Akkermansia* and *Eubacterium* in the HDCA-supplemented groups is consistent with a potential association between HDCA supplementation and microbial taxa involved in SCFA production or bile acid transformation. However, bile acid pools, SCFAs, and microbial activity were not directly measured. Therefore, the proposed gut microbiota–bile acid mechanism remains speculative and requires targeted metabolomic and mechanistic validation. Our study detected increased relative abundance of Verrucomicrobia at the phylum level in both HFD+HDCA and GHFD+HDCA groups. At the family level, elevated abundance of Verrucomicrobiaceae was observed in both groups. Verrucomicrobiaceae is a family within the phylum Verrucomicrobiota; one of its best-characterized species is *Akkermansia muciniphila* (*A. muciniphila*), an obligate anaerobe capable of degrading intestinal mucin and producing SCFAs such as acetate and propionate [40]. The first human clinical trial reported that *A. muciniphila* supplementation improves insulin sensitivity, reduces body weight, and decreases inflammatory markers [27].

At the genus level, both HFD+HDCA and GHFD+HDCA groups showed increased abundance of *Eubacterium*. This genus consists of strictly anaerobic Gram-positive bacteria belonging to the Firmicutes phylum, widely present in human and animal intestines. As a core component of gut microbiota, *Eubacterium* helps maintain intestinal microecological balance [46]. It can ferment complex polysaccharides to produce SCFAs including butyrate, propionate, and acetate [47], and regulates host immunity and energy metabolism through its metabolites [26]. Multiple studies have found significantly reduced *Eubacterium* abundance in obese individuals and high-fat diet-fed animal models, while supplementation with *Eubacterium* or its metabolites can alleviate obesity phenotypes [48]. The co-enrichment of *Akkermansia* and *Eubacterium* in HDCA-supplemented groups suggests that HDCA may promote a gut ecosystem conducive to SCFA production and metabolic homeostasis. Although several taxa enriched in the HDCA-associated groups have previously been linked with metabolic health, the biological interpretation of genus-level microbial changes requires caution. Some taxa identified in our analysis, including *Clostridium* XVIII and *Anaerotruncus*, have been reported in both beneficial and disease-associated contexts depending on species, strain, host condition, and experimental setting. Similarly, the presence of taxa such as *Coprococcus* in HFD-associated groups does not necessarily indicate a uniformly detrimental role. Therefore, these findings should be interpreted as context-dependent microbial associations rather than definitive classification of beneficial or harmful bacteria. Similarly, taxa enriched in control groups, such as *Alistipes*, may also exhibit context-dependent associations and should not be interpreted solely as indicators of a uniformly beneficial state.

PICRUSt2-based functional prediction generated hypotheses regarding potential differences in microbial functional capacity. Pathways annotated as propionate and phosphonate metabolism showed higher predicted abundance in HFD+HDCA dams, whereas pathways annotated as pyruvate, propionate, and β-alanine metabolism showed higher predicted abundance in GHFD+HDCA offspring. These annotations may be relevant to microbial energy metabolism, but PICRUSt2 does not establish pathway activity or metabolite production. Accordingly, these findings should be regarded as hypothesis-generating until confirmed by direct quantification of bile acids, SCFAs, and other metabolites.

Several limitations should be acknowledged in this study. First, the relatively small number of animals included in each experimental group may limit the statistical power and generalizability of the findings. Future studies with larger sample sizes are needed to validate the observed associations and improve the robustness of the conclusions. Second, although 5R 16S rRNA gene sequencing provides enhanced taxonomic resolution and broader coverage compared with conventional short-region 16S sequencing, it primarily provides information on microbial composition and does not directly capture strain-level variation or functional gene activity. Therefore, the functional profiles generated by PICRUSt2 should be interpreted as predictions of microbial functional potential based on 16S rRNA gene data and reference databases, rather than evidence that the predicted metabolic pathways are actively operating or that corresponding metabolites are actually produced. This limitation is particularly relevant because our study discussed potential involvement of short-chain fatty acid (SCFA)-related pathways, including propionate metabolism. Direct quantification of SCFAs using targeted metabolomics approaches, such as GC-MS or LC-MS analysis of fecal samples, cecal contents, or plasma, will be necessary in future studies to provide direct evidence supporting these mechanistic interpretations. Third, the current study was based on observational associations between maternal dietary intervention, gut microbiota alterations, and offspring obesity related phenotypes. Although the findings suggest a potential involvement of the gut microbiota-bile acid axis, causal relationships cannot be established from the present data. Future studies using fecal microbiota transplantation, germ-free animal models, or other mechanistic approaches are required to determine whether microbiota alterations directly contribute to the observed offspring phenotypes. Fourth, offspring from the same dam share a common intrauterine environment, maternal microbiota exposure, and lactational environment, and therefore should not be considered completely independent biological replicates. According to the ARRIVE guidelines, the experimental unit should be defined as the smallest biological entity that independently receives the experimental intervention. In the present study, maternal dietary intervention was applied at the dam level; therefore, the dam/litter rather than individual offspring represents the primary experimental unit. Therefore, offspring-level analyses may be affected by potential litter effects and pseudo-replication bias. Because litter was not explicitly modeled as a random effect in the present analysis, potential litter-level dependence cannot be fully excluded. The observed differences between offspring groups should consequently be interpreted as exploratory associations rather than definitive causal effects. Previous studies have emphasized the importance of accounting for litter effects and appropriately considering litter as an experimental unit in animal studies [49,50]. Fifth, because male breeders consumed the same group-specific diets during the mating period (approximately 1–2 days), a potential paternal dietary contribution cannot be completely excluded. Although prolonged paternal nutritional exposure has been shown to influence offspring metabolic traits through epigenetic modifications [51,52], whether such short-term paternal exposure exerts substantial effects remains unclear. Future studies incorporating paternal-specific dietary intervention designs will help to better distinguish maternal and paternal contributions. Sixth, liver histology and visceral adiposity were evaluated descriptively using representative images without quantitative histological scoring, fat-depot weights, hepatic triglyceride measurements, or inflammatory markers. Therefore, our conclusions regarding hepatic steatosis, inflammatory status, and adiposity should be interpreted as morphological observations rather than quantitative evidence. Another limitation is the temporal separation between microbiota profiling and later metabolic phenotyping. Offspring gut microbiota was characterized only at weaning, whereas obesity-related phenotypes were evaluated at 20 weeks of age. Therefore, the present study cannot determine whether the microbiota differences observed at weaning persisted into adulthood or directly contributed to later metabolic outcomes. In addition, because offspring remained with HDCA-treated dams during lactation, direct exposure to HDCA or HDCA-derived metabolites through maternal milk cannot be excluded as an alternative explanation. Future studies incorporating longitudinal microbiota profiling and measurements of maternal milk components during lactation will be required to clarify these mechanisms.

Previous studies have demonstrated that maternal nutritional interventions can modulate offspring developmental outcomes, while also highlighting the complexity and context dependency of these effects. For example, maternal choline supplementation has been reported to partially alleviate obesity-related developmental alterations induced by maternal obesogenic diet exposure in rodents, with effects varying according to offspring sex and specific phenotypic outcomes [53]. Similarly, methylating micronutrient supplementation during pregnancy has been shown to influence fetal hepatic gene expression, insulin-like growth factor (IGF) signaling, and fetal growth in pigs, suggesting that the biological consequences of maternal dietary interventions may differ across species and experimental conditions [54]. These findings support the concept that maternal nutrition plays an important role in early-life programming, while emphasizing the need for cautious interpretation of specific intervention effects.

Furthermore, although animal models enable mechanistic investigation of microbiota-mediated metabolic regulation under controlled conditions, human host–microbiome interactions are shaped by multiple factors, including genetic background, lifestyle, environmental exposure, and pre-existing metabolic status. Human studies have indicated that dietary patterns are associated with microbiota-related characteristics and metabolic parameters; however, these associations do not always translate into consistent biochemical improvements, reflecting the multifactorial regulation of host–microbiome interactions [55]. The present study was conducted using a C57BL/6J mouse model, which provides a controlled experimental system to investigate the effects of maternal high-fat diet exposure and HDCA supplementation on offspring gut microbiota and metabolic phenotypes. However, differences between mice and humans, including pregnancy physiology, dietary complexity, gut microbial ecology, and bile acid metabolism, may influence the extent to which these findings can be extrapolated to human populations. Therefore, the enrichment of SCFA-producing bacteria and improvement of obesity-related phenotypes observed following HDCA supplementation in the present study should be interpreted as mechanistic evidence derived from an experimental model rather than direct evidence of clinical efficacy in humans.

Despite these limitations, the study provides correlative evidence that HDCA supplementation during maternal HFD exposure was associated with differences in dam and offspring gut microbiota and with attenuation of selected obesity-related phenotypes. HDCA warrants further investigation as a candidate dietary intervention, but litter-aware analyses, direct metabolite measurements, and causal experiments are required before mechanistic or translational conclusions can be drawn [56,57].

Collectively, these considerations highlight the importance of further validation through well-designed human cohort studies and clinical investigations. Future research integrating animal models with longitudinal human studies will be essential to determine whether maternal bile acid-based interventions can effectively modulate microbiota-mediated metabolic outcomes across generations.

## 5. Conclusions

In conclusion, HDCA supplementation during maternal HFD exposure was associated with differences in gut microbial composition in dams and offspring microbiota at weaning, together with attenuation of selected obesity-related phenotypes. Several taxa showed concordant directional changes between dams and offspring under corresponding dietary conditions, indicating similar microbiota responses associated with HFD exposure and HDCA supplementation. However, the present study does not establish direct microbial transmission, altered bile-acid metabolism, increased SCFA production, or causality. Further litter-aware, sex-aware, longitudinal, and mechanistic studies with direct metabolite measurements are required to validate these findings and clarify the underlying mechanisms.

## Figures and Tables

**Table 1:** Reported Biological Associations of Genera Identified in Maternal and Offspring Gut Microbiota.

Genus *	Introduction
*Bifidobacterium*	Reported as an early-life gut colonizer with immunomodulatory functions in some human and animal studies [26,27]. Associations are species-, strain-, and host-context dependent.
*Allobaculum*	Reported in association with leanness, butyrate production, and intestinal ANGPTL4 expression in some mouse studies [28,29].
*Ruminococcus*	A common gut commensal with roles in polysaccharide degradation; individual species and strains have also been reported in inflammatory and metabolic contexts [30].
*Coprococcus*	Reported in association with microbial metabolite profiles and neuro-behavioral phenotypes in some studies [31].
*Clostridium* XI	Members of Clostridium cluster XI have been reported in bile-acid-related and disease-associated contexts; effects may differ across species and strains [32].
*Clostridium* XIVb	Conditionally pathogenic. Peanut skin procyanidin extract (PSPE) and its fraction (PSPc) exhibit antioxidant, antimicrobial, and anticancer properties. In dextran sulfate sodium (DSS)-induced colitis mice, PSPEc treatment increased *Clostridium* XIVb and *Anaerotrunus* abundance, altering gut microbiota composition and enhancing SCFAs production [33].
*Anaerotruncus*	Conditionally pathogenic. A protective factor in NAFLD [34], yet environmental toxicant (ET) exposure links its increased abundance (with *Ruminococcus*) to obesity and insulin resistance via dysbiosis and altered bile acids [35].
*Bacteroides*	Bacteroides species have strain- and site-dependent roles in polysaccharide metabolism, host nutrition, opportunistic infection, and antimicrobial resistance [36,37].
*Akkermansia*	A mucin-associated genus investigated for links with intestinal barrier function and metabolic health. Reported effects are species-, preparation-, and host-context dependent [38,39].
*Clostridium* XVIII	Conditionally pathogenic. In vitro fermentation experiments demonstrate that *Clostridium* XVIII can produce high levels of SCFAs such as propionate and butyrate during metabolism. This bacterium may regulate gut health through SCFA metabolism, influencing host energy metabolism and immune function [40]. Increased abundance of *Clostridium* XVIII in IBS patients’ feces correlates positively with depression symptom scores, suggesting its potential involvement in gut–brain axis regulation and psychological status in IBS patients.
*Eubacterium*	Some Eubacterium species produce butyrate or participate in bile-acid and cholesterol transformations; associations vary across species and disease contexts [41].
*Parabacteroides*	Parabacteroides species have been studied in relation to immune regulation, glucose metabolism, SCFA production, and antimicrobial resistance; effects are strain- and context-dependent [42].

* Genera are grouped according to their occurrence in corresponding dam and offspring dietary groups. Reported biological associations are context-dependent and should not be interpreted as genus-wide classifications of benefit, harm, or pathogenicity.

## Data Availability

The data that support the findings of this study are openly available in Sequence Read Archive at https://www.ncbi.nlm.nih.gov/sra/PRJNA1499098, reference number PRJNA1499098 (accessed on 23 July 2026).

## Supplementary Materials

The following supporting information can be downloaded at: https://www.mdpi.com/article/10.3390/metabo16080590/s1, Figure S1: Cage-based food intake of maternal mice during dietary intervention; Table S1: Composition of the control diet and high-fat diet for mice; Table S2. Two-way repeated-measures ANOVA of maternal body weight during dietary intervention; Table S3. Linear mixed-effects model analysis of longitudinal offspring body weight; Table S4. PERMANOVA results for pairwise comparisons of gut microbial community structure based on Bray–Curtis distances. Table S5. Differentially abundant bacterial genera between maternal and offspring dietary intervention groups, as determined by ANCOM-BC2.
