# Supplementary material for: HDCA Supplementation During Maternal High-Fat Diet Exposure Is Associated with Offspring Gut Microbiota at Weaning and Adult Metabolic Phenotypes in a Mouse Model
Source: Metabolites. 2026 Aug 19;16(8):590. doi: 10.3390/metabo16080590 (PMC13515643; doi:10.3390/metabo16080590)
Supplement: Supplementary file 1 [file metabolites-16-00590-s001.zip › metabolites-4473347-supplementary.pdf]

Supplementary Table S1. Composition of the control diet and high-fat diet for mice.

Product #	Control diet（LAD3001G）	High-fat diet (TP23520)
**Ingredient**	**g/kg**	
Casein	200	258
Corn Starch	397.486	0
Maltodextrin	132	162
Sucrose	100	89
Soybean Oil	70	32
Lard	-	317
Cellulose	50	65
Mineral Mix	35	58
Vitamin Mix	10	13
L-Cystine	3.0	4
Choline Chloride	2.5	-
Choline Bitartrate	-	3
TBHQ	0.014	0.069
Total	1000	1000
**Items**	**kcal %**	
Protein	19.4	18.3
Fat	16.9	60.9
Carbohydrate	63.6	20.8
**kal/g**	3.7	5.2

Supplementary Table S2. Two-way repeated-measures ANOVA of maternal body weight during dietary intervention

Source of variation	F value	P value
Time	291.2	<0.0001
Group	24.77	<0.0001
Time × Group interaction	14.97	<0.0001

Supplementary Table S3. Linear mixed-effects model analysis of longitudinal offspring body weight

Source of variation	F value	P value
Group	10.35	0.0062
Sex	68.36	<0.001
Week	322.01	<0.0001
Group × Sex	1.38	0.260
Group × Week	2.07	0.0058
Sex × Week	27.15	<0.0001
Group × Sex × Week	0.94	0.521

Data were analyzed using a linear mixed-effects model with diet group, sex, and age (week) as fixed effects and individual mouse identity as a random effect to account for repeated measurements. P values

for fixed effects and interactions are shown.

Supplementary Table S4. PERMANOVA results for pairwise comparisons of gut microbial community structure based on Bray-Curtis distances

Level	Comparison	R^2	Pseudo-F	P-value	q value
Offspring	GHFD+HDCA vs GHFD	0.473	17.049	<0.001	<0.001
	GHFD+HDCA vs GCON	0.532	28.415	<0.001	<0.001
	GHFD vs GCON	0.481	24.070	<0.001	<0.001
Maternal	HFD+HDCA vs HFD	0.468	9.690	<0.001	0.004
	HFD+HDCA vs CON	0.670	22.328	<0.001	<0.001
	HFD vs CON	0.433	7.629	<0.001	0.003
Combined (dams + offspring)	GHFD+HDCA vs HFD+HDCA	0.368	8.728	<0.001	<0.001
	GHFD+HDCA vs HFD	0.436	10.833	<0.001	<0.001
	GHFD+HDCA vs CON	0.493	13.605	<0.001	<0.001
	GHFD vs HFD+HDCA	0.537	18.592	<0.001	<0.001
	GHFD vs HFD	0.313	6.839	<0.001	<0.001
	GHFD vs CON	0.436	11.590	<0.001	<0.001
	GCON vs HFD+HDCA	0.575	29.704	<0.001	<0.001
	GCON vs HFD	0.402	14.094	<0.001	<0.001
	GCON vs CON	0.238	6.542	<0.001	<0.001
Sex-stratified (offspring)	GCONM vs GCONF	0.238	7.526	0.976	<0.001
	GHFDM vs GHFDF	0.0731	1.597	0.087	0.087
	GHFD+HDCAM vs GHFD+HDCAF	0.0216	0.342	<0.001	0.976

PERMANOVA was performed using Bray-Curtis distances with 999 permutations. P values were adjusted for multiple comparisons using the Bonferroni correction. CON, control diet; HFD, high-fat diet; HFD+HDCA, high-fat diet supplemented with 0.5% hyodeoxycholic acid; GCON, GHFD, and GHFD+HDCA denote the corresponding offspring groups; M, male; F, female. R^2 indicates the proportion of variance explained by the group factor. The last three rows represent sex-stratified comparisons within each offspring dietary group (males vs females). $q < 0.05$ was considered statistically significant.

Supplementary Table S5. Differentially abundant bacterial genera between maternal and offspring dietary intervention groups, as determined by ANCOM-BC2.

Level	Comparison	Taxon	LogFC	SE	W	P value	q value	Variation trend
**Maternal**	HFD vs CON	*Clostridium*_XVIII	3.4300	0.4103	8.3593	<0.0001	<0.0001	↑
		Lachnospiraceae_Unknown_genus3261	4.2519	0.5027	8.4576	<0.0001	<0.0001	↑
		rc4-4	3.3934	0.6975	4.8647	0.0002	0.0005	↑
		Coprococcus	2.3765	0.5192	4.5771	0.0004	0.0015	↑
		Dorea	2.1178	0.5331	3.9728	0.0011	0.0034	↑
		Bacteria_Firmicutes_Clostridia_Clostridiales_Lachnospiraceae_Ruminococcus	1.9313	0.5643	3.4225	0.0035	0.0107	↑
		Ruminococcaceae_Unknown_genus1384	1.7839	0.5471	3.2605	0.0062	0.0190	↑
		Adlercreutzia	1.3841	0.4378	3.1617	0.0069	0.0282	↑
	HFD+HDCA vs HFD	*Parabacteroides*	3.8330	0.7013	5.4655	<0.0001	0.0002	↑
		Bifidobacterium	-2.7762	0.6965	-3.9858	0.0018	0.0056	↓
		Coprococcus	-2.1050	0.6213	-3.3882	0.0041	0.0125	↓
		Allobaculum	-1.6538	0.5872	-2.8165	0.0124	0.0381	↓
**Offspring**	GHFD vs GCON	*Coprococcus*	3.2758	0.3686	8.8877	<0.0001	<0.0001	↑
		Bacteroides	3.1063	0.3589	8.6553	<0.0001	<0.0001	↑
		Lachnospiraceae_Unknown_genus3261	3.2378	0.3825	8.4659	<0.0001	<0.0001	↑
		Lachnospiraceae_Unknown_genus3829	2.5535	0.3300	7.7379	<0.0001	<0.0001	↑
		*Clostridium*_XI	2.7511	0.3831	7.1813	<0.0001	<0.0001	↑
		*Clostridium*_XlVb	2.4251	0.3820	6.3480	<0.0001	<0.0001	↑
		Oscillospira	1.8920	0.3604	5.2501	<0.0001	<0.0001	↑
		Lachnospiraceae_Unknown_genus3056	1.8214	0.3608	5.0476	<0.0001	0.0001	↑
		Allobaculum	-2.1276	0.5409	-3.9335	0.0004	0.0012	↓

	Ruminococcaceae_Unknown_genus2821	1.8322	0.4410	4.1545	0.0005	0.0016	↑
	Bifidobacterium	-1.4525	0.4455	-3.2602	0.0026	0.0053	↓
GHFD+HDCA vs GHFD	*Bifidobacterium*	-3.3727	0.4648	-7.2565	<0.0001	<0.0001	↓
	rc4-4	-3.2587	0.4833	-6.7432	<0.0001	<0.0001	↓
	Anaerotruncus	2.9539	0.5456	5.4144	<0.0001	<0.0001	↑
	*Clostridium*_XlVb	-2.6372	0.4267	-6.1804	<0.0001	<0.0001	↓
	Coprococcus	-3.1953	0.6068	-5.2662	<0.0001	<0.0001	↓
	Barnesiella	-2.8137	0.6074	-4.6322	<0.0001	0.0002	↓
	*Clostridium*_XI	-2.2590	0.4960	-4.5540	0.0001	0.0003	↓
	Clostridiales_Unknown_family_Unknown_genus3006	-2.1308	0.5818	-3.6626	0.0017	0.0051	↓
	Allobaculum	-2.3362	0.8423	-2.7735	0.0089	0.0179	↓
	Oscillospira	-2.1688	0.7650	-2.8352	0.0076	0.0227	↓
	Adlercreutzia	1.3274	0.4839	2.7434	0.0100	0.0368	↑

ANCOM-BC2 analysis was performed to identify differentially abundant genera between pairwisegroup comparisons. Only taxa with a significant q value ($q < 0.05$) are shown. Variation trend: 1, increased abundance in the first grouprelative to the second; decreased abundance in the first group relative to the second. CON, control diet; HFD, high-fat diet; HFD+HDCA, high-fat diet supplemented with 0.5% hyodeoxycholic acid; GCON, GHFD, and GHFD+HDCA denote the corresponding offspring groups

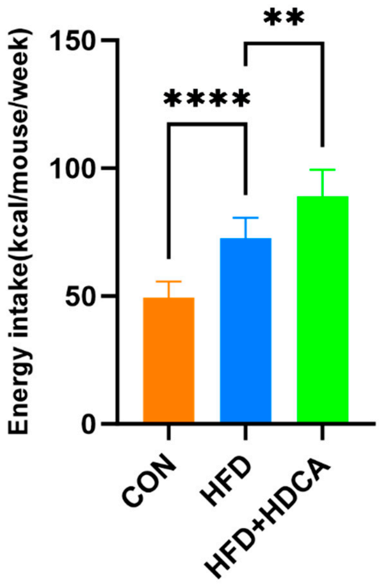

Supplementary Figure S1. Cage-based food intake of maternal mice during dietary intervention. Food consumption was recorded weekly at the cage level from 5 to 13 weeks of age in the CON, HFD, and HFD+HDCA groups. Mice were housed in cages, and weekly food intake was calculated by dividing the amount of consumed diet per cage by the number of mice in each cage. Data are presented as mean ± SEM of cages. CON, control diet; HFD, high-fat diet; HFD+HDCA, high-fat diet supplemented with HDCA. ns, not significant; *P < 0.05, **P < 0.01, ***P < 0.001, ****P < 0.0001.
